# Hearing a Feeling: Music Emotion Recognition and Callous‐Unemotional Traits in Early Childhood

**DOI:** 10.1111/cdev.70024

**Published:** 2025-08-04

**Authors:** Yael Paz, Sydney Sun, Michaela Flum, Yuheiry Rodriguez, Erin Brown, Rista C. Plate, Rebecca Waller

**Affiliations:** ^1^ Department of Psychology University of Pennsylvania Philadelphia Pennsylvania USA

**Keywords:** emotion categorization, emotion development, emotion perception, music

## Abstract

Music is a powerful medium to study emotion recognition. However, findings are mixed regarding the proficiency of young children to detect emotion conveyed by music. Moreover, we lack knowledge about music emotion recognition and callous‐unemotional traits, which portend risk for externalizing problems. The current study examined the performance of 144 children aged 3–5 years old (47.9% female; 34.0% minoritized race/ethnicity) during a music recognition task, with clips conveying happiness, sadness, calmness, or fear. Children showed above‐chance accuracy, particularly for high‐arousal emotions (happiness, fear), with accuracy increasing from 3 to 5 years old. Children higher on callous‐unemotional traits showed poorer emotion recognition, particularly for positively valenced music. Findings underscore the potential for music to promote emotion recognition and social competence skills across development.

Recognizing and responding appropriately to emotion is vital to social functioning. Music is a powerful medium that conveys emotion (Juslin and Laukka 2004), commonly through social activities (e.g., dancing, singing) and media (e.g., movies, advertisements) (Schubert 2013). Importantly, music can bypass experience and culture to *evoke* and elicit an embodied sense of emotion, with musical properties universal in their communicative function (e.g., tempo, volume) (Balkwill et al. 2004; Gregory and Varney 1996; Vuoskoski and Eerola 2011). Thus, music represents an important heuristic through which to understand emotion recognition (Eerola and Vuoskoski 2013; Timmers 2017).

Broader emotion recognition (e.g., facial, bodily posture) is commonly studied using the circumplex model, which includes valence (negative to positive) and arousal (low to high) dimensions (Russell et al. 1980). Children first recognize the valence of facial expressions (Leppänen et al. 2018; Nichols et al. 2015; Posner et al. 2005; Vaillant‐Molina et al. 2013; Wellman et al. 1995). Preschool children can differentiate happy and sad faces (different valence), but confuse sad and angry faces (same valence, different arousal) (Durand et al. 2007; Gao and Maurer 2010; Widen 2013). Across development, with input from the social environment and reinforcement of emotion categories (Denham et al. 1994), children understand specific emotions, successfully differentiating both valence and arousal (Belmon et al. 2023). Music, with its direct and potentially evocative conveyance of emotion, therefore represents a useful paradigm to characterize early emotion knowledge (Herba et al. 2006; Montirosso et al. 2010), particularly for preschool children who are still learning to recognize facial cues to emotion and/or verbally convey their feelings.

In relation to music, infants distinguish cues that are relevant to emotion, including pitch (i.e., highness or lowness of sounds; He and Trainor 2009), consonance and dissonance (i.e., pleasantness vs. unpleasantness of the intervals of two or more notes heard simultaneously; Trainor et al. 2002; Zentner and Kagan 1998) and tempo (i.e., speed; Hannon and Johnson 2005), while sensitivity to tonality, harmony, and key (i.e., melody around a central note and scale) improves across childhood (Hannon and Trainor 2007; Kastner and Crowder 1990). Children show increasing accuracy in recognizing specific emotions in music from 5 to 11 years old (Andrade et al. 2016; Hunter et al. 2011; Kragness et al. 2022; Vidas et al. 2018). Similar to studies of emotional faces (Mancini et al. 2013), recognition of sad music is learned more gradually (Plate et al. 2024; Stachó et al. 2013). However, findings are mixed for preschool‐aged children, with some studies reporting limited accuracy in recognizing emotions in music (Dalla Bella et al. 2001; Gregory et al. 1996), or above‐chance accuracy only for specific emotions (e.g., happiness; Franco et al. 2017). Thus, more studies are needed in the preschool period, including studies that leverage developmentally appropriate tasks to assess accuracy (Kastner and Crowder 1990). Moreover, studies need to investigate both emotion‐based dimensions (e.g., arousal, valence) and music properties (e.g., tempo, key) that could give greater insight into young children's ability to recognize the emotion conveyed by music.

Studies of emotion recognition in young children, including those leveraging music, can also inform our knowledge of both adaptive (Denham 2006; Eggum et al. 2011) and maladaptive (Easter et al. 2005; Fine et al. 2003) trajectories of social development. For example, children with callous‐unemotional (CU) traits (i.e., low empathy, limited guilt) are at risk for severe externalizing problems, including aggression and rule‐breaking (Frick et al. 2014; Longman et al. 2016; Waller et al. 2020). Currently, we lack effective, personalized treatments for CU traits, which could reduce associated harmful trajectories of externalizing psychopathology by targeting emotion recognition through adjunctive therapeutic efforts (Perlstein, Fair, et al. 2023).

CU traits are thought to arise from low threat sensitivity (i.e., reduced behavioral, physiological, or neural responses to threat), which encompasses difficulties recognizing facial and bodily expressions that convey fear, sadness, or anger (Kimonis et al. 2016; Powell et al. 2023; White et al. 2016) and stymies children's ability to learn that their behavior is harmful (Waller and Wagner 2019). However, no studies have tested whether CU traits are related to difficulties recognizing threat from music, which conveys emotion in evocative ways that might overcome purported neural or physiological hyperarousal. CU traits are also thought to arise from low affiliation, defined as deficient motivation and enjoyment of social closeness, with supportive evidence from prior studies examining children's responses to social bonding cues (e.g., laughter, happy faces, social imitation) (Kimonis et al. 2023; Wagner et al. 2020). However, studies have not examined whether CU traits are related to difficulties recognizing positively valenced *music*, a universal social bonding cue (Juslin and Laukka 2004). Knowledge about music emotion recognition could inform our understanding of risk mechanisms underlying the development of CU traits, including sensitivity to threat and affiliation.

In the current preregistered study, we examined music emotion recognition in 3–5‐year‐old children and tested associations with CU traits. In line with prior studies (Andrade et al. 2016; Vidas et al. 2018), we used a forced‐choice task where children heard validated instrumental music clips conveying happiness, sadness, fear, or calmness and matched them to an emotional face. For our first aim, we examined music emotion recognition accuracy, focusing on how accuracy related to valence, arousal, and musical features of clips, as well as age and sex, with prior studies reporting greater emotion recognition accuracy by girls for instrumental music (Franco et al. 2017). We hypothesized that children would show above‐chance accuracy across emotion types, with increasing accuracy across ages 3–5. For our second aim, we examined accuracy as a function of CU traits, hypothesizing that children with higher CU traits would show lower overall task accuracy and particularly prominent difficulties recognizing fearful, sad, and happy music. We focused on a community sample since both CU traits and CP are dimensional constructs, and even children with higher levels of CU traits within community samples are at risk for poorer educational, behavioral, and interpersonal outcomes (Frick et al. 2014). Moreover, use of a community sample can help to isolate specific socioemotional processes related to risk for CU traits (and not necessarily confounded by other comorbid difficulties present in clinic‐referred samples), including in ways that inform preventative interventions. Finally, we hypothesized that recognition difficulties would be driven by the temperamental features underlying CU traits, including lower threat sensitivity mapping onto poorer recognition of fearful and sad music and lower affiliation mapping onto poorer recognition of happy music (Waller and Wagner 2019).

## 1 Method

### 1.1 Participants

Data were from the online Preschool Emotional and Social Stimulus (PRESS) study, which recruited 162 children and a parent between February 2023 and February 2024, primarily from the Northeastern United States (child age in months, M = 53.02, SD = 9.90, *range* = 36–71; female, *n* = 81, 50.0%; Asian, *n* = 10, 6.2%; Black, *n* = 20, 12.3%; White, *n* = 108, 66.7%; more than one race, *n* = 24, 14.8%; see Methods, S1). The current analyses included 144 participants (age 3, *n* = 49, 34.0%; age 4, *n* = 48, 33.33%; age 5, *n* = 47, 32.6%; female, *n* = 69, 47.9%). Of the original 162, we excluded children for technical difficulties (*n =* 8), behavioral difficulties during the task (*n =* 2), having < 75% of trials (*n =* 2), or patterned response styles (*n =* 6).

### 1.2 Power Analyses

Sample size was determined ahead of preregistration (https://aspredicted.org/Y5Z_JD2) using the “pwr.anova.test” function from the “pwr” package in R (Champley 2020).

### 1.3 Design and Procedure

Participants were recruited through *Meta* with the geographic location restricted to the wider metropolitan area of Philadelphia, flyers posted in daycares, playgrounds, and stores, and an institutionally maintained database. Parents and children participated in a 30‐min Zoom call with a research assistant. We obtained written consent from parents and verbal assent from children. Parents were emailed a link to complete questionnaires. Families were compensated $50 for completing the visit and $20 for questionnaires. Study procedures were approved by the Institutional Review Board at the University of Pennsylvania.

### 1.4 Measures

#### 1.4.1 Music Emotion Listening Task (MELT)

We used an abbreviated version of the Music Emotion Listening Task (MELT) (Plate et al. 2024; Plate, Jones, et al. 2023), which we adapted for young children. Similar to prior studies of adults (Bigliassi et al. 2015; Eerola and Vuoskoski 2010) and children (Nawrot 2003; Quintin et al. 2011), we included 20 instrumental (i.e., no lyrics) 5 s music clips, which conveyed fear (negative valence, high arousal), sadness (negative valence, low arousal), happiness (positive valence, high arousal), or calmness (positive valence, low arousal) (5 clips for each) (Russell et al. 1980). To advance knowledge about objective music characteristics related to recognition accuracy, we extracted information about the key and tempo of each music clip using online software (https://tunebat.com/Analyzer).

The research assistant completed an audio check to ensure that children could hear the clips by having them repeat the word “apple.” Children were shown the response options, which were drawings of facial expressions conveying the emotions (Figure S1; Chester et al. 2022). Children had to identify which face expressed what emotion by pointing to the correct emotional face or naming the color of the face (black, red, green, and blue). Before the task, the research assistant reinforced the response options by naming the colors and emotions together (e.g., green is happy). Children were told that they would hear different music clips and would say which face matched the music by pointing to the face or naming its color. There was one practice trial. Children were then presented with 20 music clips randomized across participants. The colors and order of the face response options were fixed within participants (i.e., same order of faces depicted in the same colors each time), but colors/order were pseudorandomized between participants (Figure S1). Consistent with prior developmental studies of emotion recognition (e.g., Denham 1986), child responses were recoded after the visit as correct (i.e., exact emotion = 2), valence‐matched only (i.e., positive or negative = 1), or incorrect/do not know (i.e., incorrect = 0). For comparability with prior studies of music, we also created a binary response as correct (1) or incorrect (0), with incorrect encompassing valence‐matched only. The convergent validity of the task was evidenced in prior studies of older children (Plate et al. 2024) and by correlations between task performance and parent‐reported child emotion recognition in the current sample (see Supporting Information).

##### 1.4.1.1 Callous‐Unemotional (CU) Traits

We used parent reports on the Inventory of Callous‐Unemotional Traits (ICU; Frick 2004), which assesses callousness (e.g., “concerned about feelings of others”), uncaring (e.g., “feels bad or guilty”), and unemotionality (e.g., “expresses feelings openly”). Consistent with recommendations, we modeled ICU items as a bifactor and extracted general factor scores to represent CU traits (*α* = 0.84) (Ray and Frick 2020; Rodriguez et al. 2016) (Supporting Information).

##### 1.4.1.2 Conduct Problems (CP)

We used parent reports on the 5‐item CP scale of the Strengths and Difficulties Questionnaire (SDQ; Goodman 1997, 2001), which assesses aggressive behavior and rule‐breaking (e.g., “lies or cheats”) and has been extensively validated in preschoolers (i.e., Croft et al. 2015). We modeled items as a single factor and extracted factor scores to represent an overarching CP score (*α* = 0.51) (Supporting Information).

##### 1.4.1.3 Threat Sensitivity and Affiliative Reward

We measured threat sensitivity and affiliation using the 28‐item parent‐reported Sensitivity to Threat and Affiliative Reward Scale (STARS), with items rated on a 4‐point scale (1 = never to 4 = always), which includes a 13‐item threat (e.g., “knows I am upset by the tone of my voice”; *α* = 0.72) and 15‐item affiliation (e.g., “likes to cuddle, tickle, or play affectionately”; *α* = 0.88) subscale.

### 1.5 Analysis Plan

Analyses were conducted using mixed‐effect linear models in R (R Core Teams 2020) using tidyverse (Wickham et al. 2019), ordinal (Christensen 2022), lme4 (Bates et al. 2014), brms (Bürkner 2017), and ggplot2 (Wickham 2016). We first examined whether accuracy (incorrect vs. correct) was above chance (i.e., *t*‐test against chance = 0.25). For our first aim, we tested associations between emotional valence and arousal and recognition accuracy, controlling for age and sex. We ran mixed effects ordinal regression with accuracy as the dependent variable (e.g., incorrect = 0, valence‐matched = 1, correct = 2), which we regressed onto valence (negative = −0.5, positive = 0.5) and arousal (low = −0.5, high = 0.5), including a by‐participant random intercept. We then entered 2‐ and 3‐way interactions between valence, arousal, and age or sex (female = −0.5, male = 0.5) as relevant. We repeated this modeling approach, regressing accuracy onto musical key (minor = −0.5, major = 0.5), tempo, and participant random effects, followed by relevant age and sex interactions. For our second aim, we regressed accuracy onto CU traits, CP, valence, and arousal, controlling for age and sex. We added 2‐ and 3‐way interactions between CU traits, valence, and arousal as relevant. In supplementary analyses, we ran mixed‐effect models regressing child accuracy onto specific emotion type (i.e., fear, happy, sad, calm) instead of valence and arousal. Finally, we regressed accuracy onto threat sensitivity and affiliation. Note that results were unchanged when we repeated analyses using logistic regression defining accuracy in binary terms (i.e., incorrect = 0, correct = 1; valence‐matched coded “incorrect”) (Results S1).

### 1.6 Transparency and Data Availability Statement

The experimental task was programmed in PsychoPy (py3.10, Peirce et al. 2019). De‐identified datasets and analysis scripts are on OSF (https://osf.io/qfut5/?view_only=38204d7ac49b4622b9cd6e62d4577443). The task will be shared upon request. We report how we determined our sample size, all data exclusions, and all manipulations and measures in the study. Design and analyses were pre‐registered (https://aspredicted.org/Y5Z_JD2).

## 2 Results

See Table S1 for descriptive statistics and correlations between study variables. Scores for the measures of CU traits and CP were consistent with other studies of community samples (CU traits, M = 17.75, SD = 7.17, *range* = 3–46; CP: M = 1.27, SD = 1.31, *range* = 0–6). CU traits and CP scores did not correlate with child age, parental education, or family income.

### 2.1 Aim 1: Can Children Recognize Emotions From Music?

Overall, children accurately identified emotions conveyed by music (i.e., correct vs. incorrect) at above‐chance levels (36% trial accuracy vs. 25% chance, *t*(143) = 7.80, *p* < 0.001). Accuracy was above chance for separate emotions (fear, 39.8%, happy, 40.1%, sad, 30.9%, calm, 33.0%; range, *t* = 3.04–6.68, df = 143, *p*s < 0.003). Combined accuracy was 59% when we included trials where children only identified the valence (valence‐matched, 23% trials). When children were incorrect, their most likely response was one that matched the valence of the correct response (Table S2; Figure S2).

**FIGURE 1:**
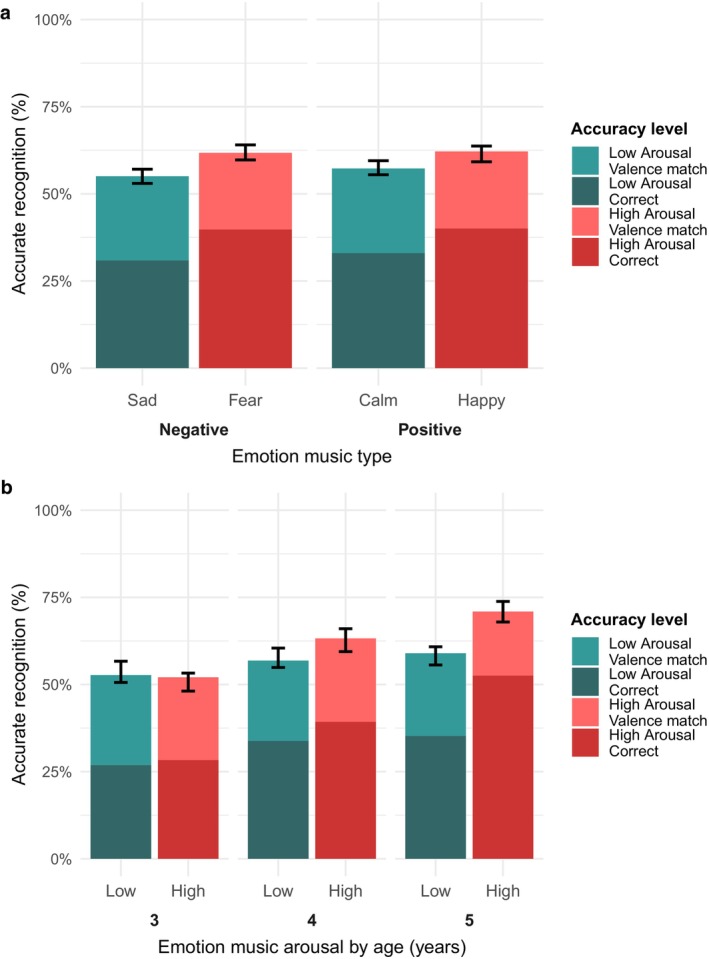
Arousal is related to music emotion recognition accuracy. (a) Emotion recognition accuracy was higher for high (happy and fear) versus low (calm and sad) arousal emotions. (b) Accuracy increased from ages 3–5, particularly for high arousal emotions.

First, accuracy was significantly better for high (i.e., happy and fear) vs. low (i.e., sad and calm) arousal clips (*B* = 0.31, SE = 0.07, OR = 1.37, *p* < 0.001; Table 1, Figure 1a). Neither valence nor the interaction of arousal and valence was related to accuracy. Second, older children showed greater accuracy for music emotion recognition (*B* = 0.29, SE = 0.05, OR = 1.33, *p* < 0.001), though sex was unrelated to accuracy (Table S3). Third, age and arousal significantly interacted (*B* = 0.27, SE = 0.07, OR = 1.32, *p* < 0.001), such that with increasing age, children became more accurate for high (*β* = 0.43, *p* < 0.001) vs. low (*β* = 0.15, *p* = 0.02) arousal music (Figure 1b). Results were similar for the specific emotions (Table S4, Figure S3). Finally, in terms of objective characteristics, children were better at recognizing emotional music clips in a major key (*B* = 0.20, SE = 0.07, OR = 1.22, *p* = 0.008; Figure S4). There was no main effect of music tempo and no interaction between key and tempo in relation to accuracy, nor differences based on age or sex (Table S5).

**TABLE 1:** Association between valence and arousal and music emotion recognition accuracy.

Predictor	Model 1			Model 2			Model 3			Model 4		
	*B*	SE	*p*	*B*	SE	*p*	*B*	SE	*p*	*B*	SE	*p*
Valence	0.05	0.07	0.473	0.05	0.07	0.473	0.05	0.07	0.489	0.05	0.07	0.480
Arousal	0.31	0.07	< 0.001	0.31	0.07	< 0.001	0.31	0.07	< 0.001	0.31	0.07	< 0.001
Valence × Arousal				−0.08	0.14	0.587	−0.08	0.14	0.595	−0.08	0.14	0.572
Child age							0.29	0.05	< 0.001	0.29	0.05	< 0.001
Child sex							−0.10	0.10	0.335	−0.10	0.11	0.343
Valence × age										−0.06	0.07	0.377
Arousal × age										0.27	0.07	< 0.001
Valence × Arousal × age										0.17	0.14	0.236

*Note:* Ordinal dependent variable: 0 = incorrect, 1 = valence recognition only, 2 = exact recognition. Emotional valence: negative = −0.5 and positive = 0.5. Arousal level: low = −0.5 and high = 0.5. Child sex: female = −0.5 and male = 0.5. Age was standardized. Results remained the same when a by‐clip random effect was entered in the model (see Table S13).

### 2.2 Aim 2: Do CU Traits Relate to Emotion Music Recognition?

Children with higher CU traits show poorer overall accuracy for recognizing emotion in music (*B* = −0.17, SE = 0.06, OR = 0.84, *p* = 0.004; Table 2). We used multinomial regression to probe this effect, showing that CU traits were related to poorer accuracy when comparing incorrect vs. correct trials (*B* = −0.21, SE = 0.07, OR = 0.80, *p* = 0.002) and incorrect vs. valence‐matched trials (*B* = −0.12, SE = 0.06, OR = 0.89, *p* = 0.05), but not valence‐matched vs. correct trials (*p* = 0.11) (Table S6).

**TABLE 2:** Associations between CU traits, conduct problems, and music emotion recognition accuracy.

Predictor	Model 1			Model 2		
	*B*	SE	*p*	*B*	SE	*p*
Emotional valence	0.05	0.07	498	0.04	0.07	0.581
Arousal level	0.31	0.07	< 0.001	0.32	0.07	< 0.001
Valence × Arousal	−0.07	0.14	0.604	−0.08	0.14	0.587
Child age	0.29	0.05	< 0.001	0.29	0.05	< 0.001
Child sex	−0.07	0.10	0.486	−0.07	0.11	0.481
Child CU traits	−0.17	0.06	0.004	−0.18	0.06	0.004
Child CP	0.03	0.06	0.603	0.03	0.06	0.595
CU traits × Valence				−0.14	0.07	0.044
CU traits × Arousal				0.15	0.07	0.039
CU traits × Valence × Arousal				−0.14	0.14	0.332

*Note:* Ordinal outcome: 0 = incorrect, 1 = valence recognition only, 2 = exact recognition. Emotional valence: negative = −0.5 and positive = 0.5. Arousal level: low = −0.5 and high = 0.5. Child sex: female = −0.5 and male = 0.5. Age, CU traits and CP were standardized. Results remained the same when a by‐clip random effect was entered in the model (see Table S14).

Abbreviations: CP, conduct problems; CU, Callous‐unemotional.

**FIGURE 2:**
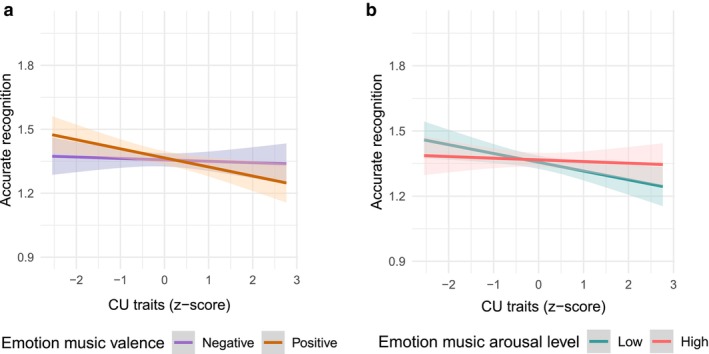
CU traits are related to worse emotion music recognition, particularly for positive and low arousal emotions. (a) CU traits were related to worse recognition of positive (*β* = −0.25, *p* < 0.001) than negative (*β* = −0.10, *p* = 0.15) emotional music. (b) CU traits were related to worse recognition of low (*β* = −0.25, *p* < 0.001) versus high (*β* = −0.10, *p* = 0.15) arousal emotions.

There was an interaction between CU traits and valence (*B* = −0.14, SE = 0.07, OR = 0.87, *p* = 0.04; Table 2), with CU traits related to poorer recognition accuracy for positively‐ (*β* = −0.25, *p* < 0.001) vs. negatively‐valenced (*β* = −0.10, *p* = 0.15) music (Figure 2a). There was also an interaction between CU traits and arousal (*B* = 0.15, SE = 0.07, OR = 1.16, *p* = 0.04), with CU traits related to poorer recognition for low (*β* = −0.25, *p* < 0.001) vs. high (*β* = −0.10, *p* = 0.15) arousal music (Figure 2b). Results were similar separating by emotion type (Table S7, Figure S5). Overall, higher CU traits were correlated with fewer correct trials for calm (*r* = −0.27, *p* = 0.001) and sad (*r* = −0.17, *p* = 0.04) music, but were unrelated to recognition for happy (*r* = −0.16, *p* = 0.06) and fearful (*r* = 0.03, *p* = 0.68) music (Table S8). There were no significant 2‐way interactions between CU traits and objective music characteristics (Table S9), age (Table S10a), or sex (Table S10b) in relation to accuracy. Results were unchanged if we did not control for co‐occurring CP (Table S11). Finally, lower threat sensitivity (*B* = −0.12, SE = 0.06, OR = 1.13, *p* = 0.04), but not lower affiliation (*p* = 0.73), was related to poorer emotion recognition accuracy (Table S12).

## 3 Discussion

We examined whether 3–5‐year‐olds could detect emotions conveyed by music, as well as variability in accuracy based on arousal and valence, and objective music properties. Overall, consistent with prior literature (Franco et al. 2017; Kastner and Crowder 1990) young children performed at above‐chance levels to identify emotions from brief music clips, though accuracy rates were low overall. Like prior studies (Andrade et al. 2016; Plate et al. 2024), accuracy increased with age, such that 5‐year‐olds outperformed 3‐year‐olds across all emotions. Findings mirror the developmental literature on emotion recognition more broadly, where children show an increasing ability to differentiate between basic emotions across the preschool period (Denham et al. 2009). However, our accuracy rates were lower than those reported by studies using facial recognition tasks at similar ages, perhaps indicating a lack of familiarity with being asked about emotion conveyed by music. Moreover, in contrast to studies of facial emotion recognition, the increasing accuracy associated with age was related to the arousal, not valence, of the music (Hunter et al. 2011). However, while there was no effect of valence on accuracy based on our a priori designations of music clips, music clips objectively identified as being in a major key (i.e., 38% of happy clips, 69% of positively valenced) were more accurately recognized. The major key is represented by specific notes and chords that evoke feelings of happiness in listeners (Hunter et al. 2010; Thompson and Cuddy 1997), with greater recognition accuracy reported for happy music in a prior study of preschoolers (Franco et al. 2017). Together, findings suggest that emotion recognition accuracy in young children is enhanced when music is stronger in arousal and/or in a major key, possibly underpinned by the greater evocative effects of clips with these properties (Coutinho and Cangelosi 2011; Franco et al. 2017; Theurel et al. 2016).

Second, within a community sample of children with overall low levels of CU traits, those children with higher CU traits showed poorer recognition of emotions in music, which is consistent with prior studies of facial, bodily, and verbal cues of emotion (Cooper et al. 2020; Powell et al. 2023; Van Zonneveld et al. 2019). That is, CU traits appear to designate a generalized difficulty in perceiving the conveyed emotion, which we show extends to the medium of music. Notably, children higher on CU traits showed difficulties simply recognizing the *valence* of music, which may have underpinned their overall emotion recognition difficulties. In addition, CU traits were more strongly related to difficulties recognizing positively valenced music. This finding is interesting when considering music as a tool that often promotes social bonding and connectedness (Nummenmaa et al. 2021; Tarr et al. 2014), though future studies are needed to investigate the mechanisms underlying any such difficulty in children with high CU traits.

Some prior work on facial emotions and CU traits has emphasized difficulties in fear recognition (Powell et al. 2023; White et al. 2016). However, in contrast to our hypotheses, CU traits were unrelated to the number of correct fearful music trials (high arousal), but did correlate with fewer correct trials for sad and calm music (low arousal). It could be that the more highly arousing music helped children “feel” the correct emotion, even if they may not have recognized it when only presented with a facial or verbal cue to emotion (cf., Hans Christian Anderson, “where words fail, music speaks”). This difficulty could result from lower activation in the audio‐motor brain regions (Koelsch 2014; Krishnan et al. 2018), as reported among adolescents with CP and CU traits (O'Nions et al. 2017). Thus, music could be used as an adjunctive tool to increase the salience of emotional cues or promote social bonding or empathic behavior (Carr et al. 2013; McDonald et al. 2022) for children high on CU traits, though this assertion would need to be tested in studies of clinic‐referred samples. Notably, our task does not disentangle the mechanisms underlying children's performance, including whether their accuracy (recognizing the emotion) is a consequence of their felt emotion (i.e., evoked by the music); these processes overlap conceptually, but are not completely aligned (Schubert 2013). Future research incorporating multi‐method assessment of recognition and resonance with emotion conveyed by music can inform greater knowledge of the socioemotional difficulties underpinning CU traits, including those related to cognitive versus affective empathy (Waller et al. 2020).

The current study had several limitations. First, the internal consistency of the CP measure was below the level considered acceptable, though use of factor scores helped to better model variance in CP, while minimizing error and increasing reliability (McNeish 2023). Second, we recruited a convenience sample with relatively low CU traits and CP, as well as many parents with high educational attainment and family income, reducing the representativeness of our sample. To increase generalizability and explore translational implications, research needs to replicate our findings among more economically diverse samples, as well as in children with clinically significant CP, where accuracy rates may be lower. Third, children only had one practice trial, which may have impacted accuracy. Fourth, we did not include other, non‐emotional identification tasks (Plate, Woodard, and Pollak 2023), which could have allowed us to address whether there was specificity in task‐related performance difficulties and CU traits. Finally, we only examined four emotions using brief music clips, emphasizing the need for future studies that can evaluate a wider range of emotions (e.g., anger, pride) and/or longer clips that change in the emotion or intensity conveyed over time. Pairing longer clips with different measurement modalities (e.g., peripheral nervous system) could provide insight into children's resonance or engagement with music (Chaturvedi et al. 2022), which may be particularly relevant for CU traits (Jones et al. 2010; Plate, Jones, et al. 2023).

In sum, preschoolers accurately recognized emotion conveyed through music, especially highly arousing emotions, while CU traits were associated with poorer recognition accuracy. We demonstrated the validity of an online assessment of children's emotion knowledge using brief music clips, expanding the accessibility of future studies evaluating early social cognition. Some emotion recognition difficulties faced by children with CU traits may be overcome when music is the medium through which emotion is conveyed, which could lay the foundation for future intervention efforts.

## Supporting information


**Table S1:** Descriptive statistics and correlation.
**Table S2:** Response distribution matrix for emotion stimuli and child emotion response selection.
**Table S3:** Association between child sex, valence, and arousal and music emotion recognition accuracy.
**Table S4:** Mixed‐effect ordinal regression analyses examining whether the emotional type predicts the accuracy of emotion recognition from music.
**Table S5:** Mixed‐effect ordinal regression analyses examining whether objective music characteristics predict the accuracy of emotion recognition from music.
**Table S6:** Mixed‐effect multinominal regression analyses probing the main effect of CU traits on the prediction of emotion recognition from music.
**Table S7:** Mixed‐effect ordinal regression analyses examining whether CU traits and conduct problems predict the accuracy of emotion recognition from music.
**Table S8:** Correlation matrix between CU traits and emotion recognition accuracy by emotion and accuracy type.
**Table S9:** Mixed‐effect ordinal regression analyses examining whether objective music characteristics predict the accuracy of emotion recognition from music.
**Table S10:** Mixed‐effect ordinal regression analyses examining whether CU traits interact with child age or sex predict the accuracy of emotion recognition from music.
**Table S11:** Mixed‐effect ordinal regression analyses examining whether CU traits predict the accuracy of emotion recognition from music, without controlling for conduct problem levels.
**Table S12:** Mixed‐effect ordinal regression analyses examining whether threat sensitivity and affiliative reward predict the accuracy of emotion recognition from music.
**Table S13:** Association between valence and arousal and music emotion recognition accuracy, controlling for by‐clip random effect.
**Table S14:** Associations between CU traits, conduct problems, and music emotion recognition accuracy, controlling for by‐clip random effect.
**Table S15:** Bifactor model of the Inventory of Callous‐Unemotional Traits (ICU) items to estimate a general CU traits factor score.
**Table S16:** Latent factor model to extract the general CP score from the parent report of the SDQ.
**Table S17:** Associations between the sum scores of CU traits, conduct problems, and music emotion recognition accuracy.
**Table S18:** Mixed‐effect logistic regression analyses examining whether the emotional valence and arousal level of sound predict the accuracy of emotion recognition from music.
**Table S19:** Mixed‐effect logistic regression analyses examining whether CU traits and conduct problems predict the accuracy of emotion recognition from music.
**Figure S1:** Response options for the Music Emotion Listening Task (MELT).
**Figure S2:** Children showed above‐chance accuracy in detecting the emotion conveyed by music.
**Figure S3:** Children showed increasing accuracy in detecting the emotion conveyed by music with increasing age.
**Figure S4:** Children are better at recognizing emotions in music in the key of major.
**Figure S5:** The association between CU traits and music emotion recognition accuracy varies by emotion type.
