# Supplementary material for: Hearing a Feeling: Music Emotion Recognition and Callous‐Unemotional Traits in Early Childhood
Source: Child Dev. 2025 Aug 4;96(6):2247–56. doi: 10.1111/cdev.70024 (PMC12598441; doi:10.1111/cdev.70024)
Supplement: Supplementary file 1 — Table S1: Descriptive statistics and correlation. Table S2: Response distribution matrix for emotion stimuli and child emotion response selection. Table S3: Association between child sex, valence, and arousal and music emotion recognition accuracy. Table S4: Mixed‐effect ordinal regression analyses examining whether the emotional type predicts the accuracy of emotion recognition from music. Table S5: Mixed‐effect ordinal regression analyses examining whether objective music characteristics predict the accuracy of emotion recognition from music. Table S6: Mixed‐effect multinominal regression analyses probing the main effect of CU traits on the prediction of emotion recognition from music. Table S7: Mixed‐effect ordinal regression analyses examining whether CU traits and conduct problems predict the accuracy of emotion recognition from music. Table S8: Correlation matrix between CU traits and emotion recognition accuracy by emotion and accuracy type. Table S9: Mixed‐effect ordinal regression analyses examining whether objective music characteristics predict the accuracy of emotion recognition from music. Table S10: Mixed‐effect ordinal regression analyses examining whether CU traits interact with child age or sex predict the accuracy of emotion recognition from music. Table S11: Mixed‐effect ordinal regression analyses examining whether CU traits predict the accuracy of emotion recognition from music, without controlling for conduct problem levels. Table S12: Mixed‐effect ordinal regression analyses examining whether threat sensitivity and affiliative reward predict the accuracy of emotion recognition from music. Table S13: Association between valence and arousal and music emotion recognition accuracy, controlling for by‐clip random effect. Table S14: Associations between CU traits, conduct problems, and music emotion recognition accuracy, controlling for by‐clip random effect. Table S15: Bifactor model of the Inventory of Callous‐Unemotional Traits (ICU) items to est [file CDEV-96-2247-s001.docx]

**Supplemental Materials**

**Hearing a Feeling: Music Emotion Recognition and Callous-Unemotional Traits in Early Childhood**

**Supplemental Methods**

**Participants**

Data were from the online PRESS study, which recruited 162 children and a parent, primarily from Philadelphia in the Northeastern United States (child age in months, *M*=53.02, *SD*=9.90, *range*=36–71; female, *n=*81, 50.0%; Asian, *n*=10, 6.2%; Black, *n*=20, 12.3%; White, *n*=108, 66.7%; Biracial or more than one race, *n*=24, 14.8%). Parents were biological mothers (*n*=159, 98%) or fathers (*n*=3, 2%) who reported being from the following racial groups: Asian (*n*=23; 14.2%), Black (*n*=17; 10.5%), White (*n*=115; 71.0%), and biracial or more than one race (*n*=7, 4.3%). More than half of parents (67%) reported having a graduate-level degree, 22% had a Bachelor level degree, 4% had completed some college, 3% had an Associate’s degree or technical training, 4% had a high school degree or less, and 1% declined to report. The average monthly household income was $14,224 (*SD* = $9,203). The current analyses included 144 participants (age 3, *n*=49, 34.0%; age 4, *n*=48, 33.33%; age 5, *n*=47, 32.6%; female, *n* = 69, 47.9%). Of the original 162 participants, we excluded children for technical difficulties (n=8), behavioral difficulties during the task (n=2), having <75% of trials (n=2), or patterned response styles (e.g., consistently selecting a specific emotion face; n=6). Children included in the current analyses did not differ to those excluded based on age (*t*(160)=1.45, *p*=.15), sex (*χ*^2^(1)=2.25, *p*=.13), or CU traits (*t*(160)=1.77, *p*=.08).

**Computing CU traits and CP scores**

Callous-Unemotional (CU) Traits. We used parent reports on the Inventory of Callous-Unemotional Traits (ICU; Frick, 2004), which assesses callousness (e.g., “concerned about feelings of others”), uncaring (e.g., “feels bad or guilty”), and unemotionality (e.g., “expresses feelings openly”). We used 21 of the original 24 items dropping items 3, 6 and 10, that correlated negatively with parent ratings on other items. Due to model convergence issues arising from several items missing ratings for the highest rating on the scale, we recoded the original 4-point scale (0=not at all true, 1=somewhat true, 2=very true, 3=definitely true) to be on a 3-point scale (0=not at all true, 1=somewhat true, 2=very true or definitely true). Next, consistent with recommendations, we modeled the ICU items as a bifactor model (Ray & Frick, 2020; Rodriguez et al., 2016), with items loading simultaneously onto an overarching general factor score representing shared variance across all items relating to CU traits, as well as three specific factors representing unique variance relating to the subfactors of callousness, uncaring, and unemotional. We extracted general factor scores for use in subsequent analyses. The model showed acceptable fit to the data *Χ*^2^(166)=245.16, *p*<.001, CFI=.94, TLI=.92, RMSEA=.054, SRMR=.093 (see **Table S15** for individual item-factor loadings).

Conduct Problems (CP). We used parent reports on the 5-item CP scale of the Strengths and Difficulties Questionnaire (SDQ; Goodman, 1997, 2001), which assesses aggressive behavior and rule-breaking (e.g., “lies or cheats”). We recoded the original 3-point scale (0=not true, 2=certainly true) to a binary scale given the low frequency of parent endorsing items as “certainly true,” which caused model estimation problems. We modeled items as a single factor and extracted factor scores to represent an overarching conduct problems score. The model showed excellent fit to the data *Χ*^2^(5)=3.34, *p*=.647, CFI= 1.00, TLI=1.00, RMSEA=.000, SRMR=.064 (see **Table S16** for individual item-factor loadings).

Note that we repeated the main analyses (**Table 2**) using the summed score of the original 24 ICU items coded on the original 4-point scale and the summed score of the 5 SDQ conduct problems items on the original 3-point scale (i.e., instead of using factor scores). Overall, the pattern of results remained similar (see **Table S17**).

**Validating MELT task**

The Music Emotion Listening Task (MELT) used in the current study was validated in a sample of 5-6 year olds (Plate, Jones, Steinberg, et al., 2023). In this prior study, we showed that greater accuracy in emotion recognition was associated with higher levels of emotion verbal expressivity (more emotion words) but not general verbal fluency. In addition, children who had greater emotion recognition reported on higher emotional resonance with the music, ability associated with parent-reported empathy. To validate the task on the current sample we examined the convergence between children accuracy task and parent report on children’s emotion recognition using specific items included within the Sensitivity to Threat and Affiliative Reward Scales (STARS) (Perlstein et al., 2023): item 4, *“Notices if a child around him/her is scared”* and item 13, *“Notices if a child around him/her is scared”* (averaged to index “fear recognition”) and item 16, *“Notices loud or aggressive people on the street”* and item 25, *“Knows if I am angry or upset by the tone of my voice”*) (averaged to index “anger/aggression recognition”). Children’s overall accuracy on the task was specifically correlated with parent-reported fear recognition (*r*=.26, *p*=.002), anger/aggression recognition (*r*=.23, *p*=.006), and the average of parent ratings across both the fear and anger/aggression scales (*r*=.29, *p*<.001). While these correlations provide support for the convergent validity of the task, the magnitude of the effect sizes is low-to-moderate. One possibility is that the STARS items assess awareness of or attention to emotions, whereas our music recognition task involved additional elements of resonance and evoking of emotion, which could have supported recognition.

**Supplemental Results**

**Aim 1: Can children recognize emotions from music?**

Using logistic regression models, where valence-matched responses were coded as incorrect, we found the same pattern of analyses as our main analyses. First, we found a main effect of arousal (*B*=.38, *SE*=.08, *p*<.001) such that children were better at recognizing high versus low arousal emotional music, with no effect of valence, nor an interaction between valence and arousal. As before, there was also a main effect of age (*B*=.32, *SE*=.06, *p*<.001), as well as an age-by-arousal interaction (*B*=.29, *SE*=.08, *p*<.001) (**Table S18**). That is, with increasing age, children became more accurate for high versus low arousal music clips.

**Aim 2: Are CU traits related to the recognition of emotion in music?**

Using logistic regression models, where valence-matched responses were coded as incorrect, we found that CU traits were related to lower overall accuracy (*B*=-.17, *SE*=.06, *p*=.013; **Table S19**). However, neither interaction between CU traits and emotion valence or arousal were significant.

| **Table S1.** Descriptive statistics and correlation | | | | | | | | | | | |
| --- | --- | --- | --- | --- | --- | --- | --- | --- | --- | --- | --- |
| *Predictor* | **Descriptive** | | | **Correlations** | | | | | | | |
|  | *M* | *sd* | *range* | *1* | *2* | *3* | *4* | *5* | *6* | *7* | *8* |
| 1. Child age | 53.02 | 9.90 | 36-71 | - |  |  |  |  |  |  |  |
| 2. Child Sex | .00 | .50 | -.50-.50 | .09 | - |  |  |  |  |  |  |
| 3. Family income | 14,224 | 9,203 | 1,250-62,500 | .15 | .11 | - |  |  |  |  |  |
| 4. Parental education | 5.43 | 1.04 | 1-6 | .09 | -.07 | .31^***^ | - |  |  |  |  |
| 5. Child CU traits | 17.75 | 7.17 | 3-46 | .02 | .07 | -.06 | -.04 | - |  |  |  |
| 6. Child CP | 1.27 | 1.31 | 0-6 | .04 | .02 | -.11 | -.10 | .57^***^ | - |  |  |
| 7. Child threat sensitivity | 3.59 | .38 | 2.62-4.77 | .16^*^ | .08 | .01 | .11 | -.37^***^ | -.17^*^ | - |  |
| 8. Childe affiliative reward | 4.44 | .42 | 3.13-5 | -.09 | -.13 | .12 | .10 | -.60^***^ | -.35^***^ | .39^***^ | - |
| CU=Callous-unemotional, CP=conduct problems; Child sex: female=-.5 and male=.5. Parent reported on monthly family income. Parental education was reported on 6-points scale ranging between 1-“Less than high school diploma” and 6- Graduate school degree“”. CU traits and CP score are sum scores, threat sensitivity and affiliative reward are item average. | | | | | | | | | | | |

| **Table S2.** Response distribution matrix for emotion stimuli and child emotion response selection | | | | | | | |
| --- | --- | --- | --- | --- | --- | --- | --- |
|  |  | **Child emotion response selection** | | | |  |  |
| **Emotion stimuli** |  | Fear | Happy | Sad | Calm | *Χ^2^(2)* | *P* |
| Fear | *N* of trials | **284** | 138 | 157 | 116 | 6.15 | .046 |
|  | % overall responses | 41% | 20% | 23% | 17% |  |  |
|  | % of incorrect responses |  | 34% | 38% | 28% |  |  |
|  | Δ Expected |  | +1 | +20 | -21 |  |  |
|  | Diff. Sq./ Exp Fr. |  | .01 | 2.92 | 3.22 |  |  |
| Happy | *N* of trials | 118 | **286** | 132 | 158 | 6.06 | .048 |
|  | % overall responses | 17% | 42% | 19% | 23% |  |  |
|  | % of incorrect responses | 29% |  | 32% | 39% |  |  |
|  | Δ Expected | -18 |  | -4 | +22 |  |  |
|  | Diff. Sq./ Exp Fr. | 2.38 |  | .12 | 3.56 |  |  |
| Sad | *N* of trials | 172 | 169 | **220** | 130 | 6.99 | .030 |
|  | % overall responses | 25% | 24% | 32% | 19% |  |  |
|  | % of incorrect responses | 37% | 36% |  | 28% |  |  |
|  | Δ Expected | +15 | +12 |  | -27 |  |  |
|  | Diff. Sq./ Exp Fr. | 1.43 | .92 |  | 4.64 |  |  |
| Calm | *N* of trials | 116 | 172 | 161 | **234** | 11.34 | .003 |
|  | % overall responses | 17% | 25% | 27% | 34% |  |  |
|  | % of incorrect responses | 26% | 38% | 36% |  |  |  |
|  | Δ Expected | -33 | +22 | +11 |  |  |  |
|  | Diff. Sq./ Exp Fr. | 7.31 | 3.23 | .81 |  |  |  |
| *Note*. N of trials represent the number of trials an emotional face was chosen in response to a specific emotional category being played. Δ Expected is the delta between N of trails chosen to expected number of trials if incorrect response was random (a third of the incorrect responses). Diff. Sq./ Exp Fr. is the squared difference between expected and observed values divided by expected frequency. A separate Chi squared test for goodness of fit was calculated for each emotion category, based on the difference between the number of observed trials an emotion was chosen in response and the expected number of trials in case children were choosing the incorrect response randomly (number of incorrect responses per emotions divided by 3). Response distribution is presented at Figure S1. | | | | | | | |

| **Table S3.** Association between child sex, valence and arousal and music emotion recognition accuracy | | | | | | |
| --- | --- | --- | --- | --- | --- | --- |
| *Predictor* | **Model 1** | | | **Model 2** | | |
|  | *B* | *SE* | *p* | *B* | *SE* | *p* |
| Emotional Valence | .05 | .07 | .488 | .05 | .07 | .493 |
| Arousal level | .31 | .07 | <.001 | .32 | .07 | <.001 |
| Valence x Arousal | -.08 | .14 | .593 | -.07 | .14 | .627 |
| Child age | .29 | .05 | <.001 | .29 | .05 | <.001 |
| Child sex | -.10 | .11 | .332 | -.10 | .11 | .334 |
| Sex x age | -.12 | .11 | .245 |  |  |  |
| Sex x Valence |  |  |  | -.04 | .14 | .780 |
| Sex x Arousal |  |  |  | .17 | .14 | .243 |
| Sex x Valence x Arousal |  |  |  | .34 | .28 | .232 |
| Ordinal dependent variable: 0=incorrect, 1=valence matching, 2=exact recognition. Emotional valence: negative=-.5 and positive=.5. Arousal level: low=-.5 and high=.5. Child sex: female=-.5 and male=.5. Age was standardized. Model 1 examined the interaction between child age and sex in the prediction of accuracy. Model 2 examine the interaction of child sex with valence and arousal in the prediction of accuracy. | | | | | | |

| **Table S4.** Mixed-effect ordinal regression analyses examining whether the emotional type predicts the accuracy of emotion recognition from music. | | | | | | | | | |
| --- | --- | --- | --- | --- | --- | --- | --- | --- | --- |
| *Predictor* | **Model 1** | | | **Model 2** | | | **Model 3** | | |
|  | *B* | *SE* | *p* | *B* | *SE* | *p* | *B* | *SE* | *p* |
| Fear emotion | .26 | .10 | .010 | .26 | .10 | .010 | .26 | .10 | .010 |
| Happy emotion | .27 | .10 | .007 | .27 | .10 | .007 | .27 | .10 | .007 |
| Sad emotion | -.09 | .10 | .373 | -.09 | .10 | .387 | -.09 | .10 | .366 |
| Child age |  |  |  | .29 | .05 | <.001 | .08 | .08 | .330 |
| Child sex |  |  |  | -.10 | .11 | .335 | -.10 | .11 | .342 |
| Fear x age |  |  |  |  |  |  | .34 | .10 | <.001 |
| Happy x age |  |  |  |  |  |  | .36 | .10 | <.001 |
| Sad x age |  |  |  |  |  |  | .15 | .10 | .138 |
| Ordinal dependent variable: 0=incorrect, 1=valence matching, 2=exact recognition. Child sex: female=-0.5 and male=0.5. Age was standardized. Calm emotion was used as the reference group. | | | | | | | | | |

| **Table S5.** Mixed-effect ordinal regression analyses examining whether objective music characteristics predict the accuracy of emotion recognition from music. | | | | | | | | | | | | |
| --- | --- | --- | --- | --- | --- | --- | --- | --- | --- | --- | --- | --- |
| *Predictor* | **Model 1** | | | **Model 2** | | | **Model 3** | | | **Model 4** | | |
|  | *B* | *SE* | *p* | *B* | *SE* | *p* | *B* | *SE* | *p* | *B* | *SE* | *p* |
| Key | .20 | .07 | .007 | .20 | .07 | .008 | .20 | .07 | .009 | .20 | .07 | .004 |
| Tempo | .00 | .04 | .918 | .00 | .04 | .963 | .00 | .04 | .944 | .00 | .04 | .972 |
| Key x Tempo |  |  |  | -.08 | .07 | .280 | -.08 | .07 | .272 | -.08 | .07 | .105 |
| Child age |  |  |  |  |  |  | .29 | .05 | <.001 | .29 | .06 | <.001 |
| Child sex |  |  |  |  |  |  | -.10 | .10 | .324 | -.10 | .10 | .351 |
| Key x age |  |  |  |  |  |  |  |  |  | .01 | .07 | .925 |
| Tempo x age |  |  |  |  |  |  |  |  |  | .01 | .04 | .823 |
| Key x Tempo x age |  |  |  |  |  |  |  |  |  | -.09 | .07 | .215 |
| Ordinal outcome: 0 = incorrect, 1 = valence matching, 2 = exact recognition. Key was coded: minor=-0.5 and major=0.5. Child sex was coded female=-0.5 and male=0.5. Music tempo and children’s age were standardized. | | | | | | | | | | | | |

| **Table S6.** Mixed-effect multinominal regression analyses probing the main effect of CU traits on the prediction of emotion recognition from music. | | | | | | | | | |
| --- | --- | --- | --- | --- | --- | --- | --- | --- | --- |
| *Predictor* | **Incorrect vs. Valence-matched** | | | **Incorrect vs. Correct** | | | **Valence-matched vs. Correct** | | |
|  | *B* | *SE* | *p* | *B* | *SE* | *p* | *B* | *SE* | *p* |
| Emotional Valence | .04 | .10 | .341 | .07 | .09 | .217 | .04 | .10 | .351 |
| Arousal level | .05 | .10 | .307 | .40 | .09 | <.001 | .34 | .11 | <.001 |
| Valence x Arousal | -.04 | .20 | .382 | -.10 | .18 | .288 | -.07 | .20 | .366 |
| Child age | .07 | .06 | .096 | .36 | .06 | <.001 | .29 | .06 | <.001 |
| Child sex | -.10 | .11 | .175 | -.10 | .12 | .217 | .01 | .13 | .469 |
| Child CU traits | -.12 | .06 | .050 | -.21 | .07 | .002 | -.09 | .08 | .112 |
| Child CP | .03 | .06 | .319 | .05 | .07 | .250 | .02 | .08 | .397 |
| CU=Callous-unemotional, CP=conduct problems; Ordinal outcome: 0=incorrect, 1=valence matching, 2=exact recognition. Emotional valence: negative=-.5 and positive=.5. Arousal level: low=-.5 and high=.5. Child sex: female=-.5 and male=.5. Age, CU traits, and CP were standardized. | | | | | | | | | |

| **Table S7.** Mixed-effect ordinal regression analyses examining whether CU traits and conduct problems predict the accuracy of emotion recognition from music. | | | | | | |
| --- | --- | --- | --- | --- | --- | --- |
| *Predictor* | **Model 1** | | | **Model 2** | | |
|  | *B* | *SE* | *p* | *B* | *SE* | *p* |
| Fear emotion | .26 | .10 | .009 | .28 | .10 | .005 |
| Happy emotion | .28 | .10 | .006 | .28 | .10 | .005 |
| Sad emotion | -.09 | .10 | .398 | -.08 | .10 | .439 |
| Child age | .29 | .05 | <.001 | .29 | .05 | <.001 |
| Child sex | -.07 | .11 | .486 | -.07 | .11 | .480 |
| Child CU traits | -.17 | .06 | .004 | -.29 | .09 | .001 |
| Child CP | .03 | .06 | .603 | .03 | .06 | .595 |
| CU traits x Fear |  |  |  | .29 | .10 | .004 |
| CU traits x Happy |  |  |  | .08 | .10 | .438 |
| CU traits x Sad |  |  |  | .08 | .10 | .463 |
| CU=Callous-unemotional, CP=conduct problems; Ordinal outcome: 0=incorrect, 1=valence matching, 2=exact recognition. Emotional valence: negative=-.5 and positive=.5. Arousal level: low=-.5 and high=.5. Child sex: female=-.5 and male=.5. Age, CU traits, and CP were standardized. Calm emotion was used as the reference group. | | | | | | |

| **Table S8.** Correlation matrix between CU traits and emotion recognition accuracy by emotion and accuracy type. | | | | | | |
| --- | --- | --- | --- | --- | --- | --- |
|  | Accuracy - Ordinal | | Accuracy - Binary | | Corrected accuracy/ Unbiased hit rate- binary | |
|  | *r* | *p* | *r* | *p* | *r* | *p* |
| Fear emotion | .03 | .626 | .03 | .748 | -.05 | .537 |
| Happy emotion | -.16 | .063 | -.15 | .075 | .01 | .914 |
| Sad emotion | -.17 | .040 | -.16 | .054 | -.26 | .002 |
| Calm emotion | -.27 | .001 | -.19 | .019 | -.25 | .002 |
| CU=Callous-unemotional; Ordinal outcome: 0=incorrect, 1=valence-matched, 2=exact recognition. Binary outcome: 0=incorrect, 1=exact recognition (partial recognition of correct valence matching was coded as 0). Corrected accuracy was calculated using the binary outcome. For each emotion category we calculated hit rate (proportion of trials when a category was chosen and was the target; using the binary accuracy score), and a false alarm rate (proportion of trials when a category was chosen but wasn’t the target). Then we calculated the corrected accuracy score Hu = hit X (1- false alarm) (Wagner et al., 1993). CU traits were standardized. | | | | | | |

| **Table S9.** Mixed-effect ordinal regression analyses examining whether objective music characteristics predict the accuracy of emotion recognition from music. | | | | | | |
| --- | --- | --- | --- | --- | --- | --- |
| *Predictor* | **Model 1** | | | **Model 2** | | |
|  | *B* | *SE* | *p* | *B* | *SE* | *p* |
| Key | .20 | .08 | .009 | .09 | .03 | .010 |
| Tempo | .00 | .04 | .955 | -.01 | .04 | .764 |
| Key x Tempo | -.08 | .07 | .272 | -.04 | .04 | .221 |
| Child age | .29 | .05 | <.001 | .29 | .05 | <.001 |
| Child sex | -.08 | .11 | .471 | -.08 | .10 | .467 |
| Child CU traits | -.17 | .06 | .004 | -.16 | .06 | .007 |
| Child CP | .03 | .06 | .591 | .03 | .06 | .578 |
| CU traits x Key |  |  |  | -.02 | .04 | .662 |
| CU traits x Tempo |  |  |  | -.04 | .04 | .294 |
| CU traits x Key x Tempo |  |  |  | -.08 | .04 | .056 |
| CU=Callous-unemotional, CP=conduct problems; Ordinal outcome: 0=incorrect, 1=valence matching, 2=exact recognition. Emotional valence: negative=-.5 and positive=.5. Arousal level: low=-.5 and high=.5. Child sex: female=-.5 and male=.5. Key was coded: minor=-0.5 and major=0.5. Tempo, age, CU traits, and CP were standardized. In model 2 to help convergence, the music key and all interactions were standardized. | | | | | | |

| **Table S10.** Mixed-effect ordinal regression analyses examining whether CU traits interaction with child age or sex predict the accuracy of emotion recognition from music. | | | | | | |
| --- | --- | --- | --- | --- | --- | --- |
| *Predictor* | **CU traits interaction with child age** | | | **CU traits interaction with child sex** | | |
|  | *B* | *SE* | *p* | *B* | *SE* | *p* |
| Emotional Valence | .05 | .07 | .498 | .05 | .07 | .494 |
| Arousal level | .31 | .07 | <.001 | .31 | .07 | <.001 |
| Valence x Arousal | -.07 | .14 | .604 | -.07 | .14 | .603 |
| Child age | .29 | .05 | <.001 | .29 | .05 | <.001 |
| Child sex | -.07 | .11 | .487 | -.07 | .11 | .508 |
| Child CU traits | -.17 | .06 | .004 | -.17 | .06 | .005 |
| Child CP | .03 | .06 | .614 | .04 | .06 | .605 |
| CU traits x age/sex | .00 | .05 | .986 | .09 | .10 | .396 |
| CU=Callous-unemotional, CP=conduct problems; Ordinal outcome: 0=incorrect, 1=valence matching, 2=exact recognition. Emotional valence: negative=-.5 and positive=.5. Arousal level: low=-.5 and high=.5. Child sex: female=-.5 and male=.5. Age, CU traits, and CP were standardized. | | | | | | |

| **Table S11.** Mixed-effect ordinal regression analyses examining whether CU traits predict the accuracy of emotion recognition from music, without controlling for conduct problem levels. | | | | | | |
| --- | --- | --- | --- | --- | --- | --- |
| *Predictor* | **Model 1** | | | **Model 2** | | |
|  | *B* | *SE* | *p* | *B* | *SE* | *p* |
| Emotional Valence | .05 | .07 | .498 | .04 | .07 | .579 |
| Arousal level | .31 | .07 | <.001 | .32 | .07 | <.001 |
| Valence x Arousal | -.07 | .14 | .603 | -.08 | .14 | .585 |
| Child age | .29 | .05 | <.001 | .30 | .05 | <.001 |
| Child sex | -.07 | .11 | .488 | -.07 | .11 | .483 |
| Child CU traits | -.16 | .05 | .002 | -.16 | .05 | .002 |
| CU traits x Valence |  |  |  | -.14 | .07 | .043 |
| CU traits x Arousal |  |  |  | .15 | .07 | .039 |
| CU traits x Valence x Arousal |  |  |  | -.14 | .14 | .331 |
| CU=Callous-unemotional, CP=conduct problems; Ordinal outcome: 0=incorrect, 1=valence matching, 2=exact recognition. Emotional valence: negative=-.5 and positive=.5. Arousal level: low=-.5 and high=.5. Child sex: female=-.5 and male=.5. Age, CU traits, and CP were standardized. | | | | | | |

| **Table S12.** Mixed-effect ordinal regression analyses examining whether threat sensitivity and affiliative reward predict the accuracy of emotion recognition from music. | | | | | | |
| --- | --- | --- | --- | --- | --- | --- |
| *Predictor* | **Model 1** | | | **Model 2** | | |
|  | *B* | *SE* | *p* | *B* | *SE* | *p* |
| Emotional Valence | .05 | .07 | .493 | .05 | .07 | .493 |
| Arousal level | .31 | .07 | <.001 | .31 | .07 | <.001 |
| Valence x Arousal | -.07 | .14 | .600 | -.07 | .14 | .599 |
| Child age | .27 | .05 | <.001 | .28 | .05 | <.001 |
| Child sex | -.08 | .11 | .433 | -.08 | .11 | .441 |
| Low threat sensitivity | -.12 | .06 | .039 | -.12 | .06 | .043 |
| Low affiliative reward | -.02 | .06 | .731 | -.03 | .06 | .628 |
| Threat sensitivity x affiliative reward |  |  |  | .02 | .05 | .647 |
| CU=Callous-unemotional, CP=conduct problems; Ordinal outcome: 0=incorrect, 1=valence matching, 2=exact recognition. Emotional valence: negative=-.5 and positive=.5. Arousal level: low=-.5 and high=.5. Child sex: female=-.5 and male=.5. Age was standardized. Threat sensitivity and affiliative reward were standardized and recorded so that high scores represent low threat sensitivity and low affiliative reward, in line with hypothesized associations with CU traits. | | | | | | |

| **Table S13.** Association between valence and arousal and music emotion recognition accuracy controlling for by-clip random effect | | | | | | | | | | | | |
| --- | --- | --- | --- | --- | --- | --- | --- | --- | --- | --- | --- | --- |
| *Predictor* | **Model 1** | | | **Model 2** | | | **Model 3** | | | **Model 4** | | |
|  | *B* | *SE* | *p* | *B* | *SE* | *p* | *B* | *SE* | *P* | *B* | *SE* | *p* |
| Valence | .05 | .11 | .626 | .05 | .11 | .624 | .05 | .11 | .634 | .05 | .11 | .627 |
| Arousal | .32 | .11 | .003 | .32 | .11 | .003 | .32 | .11 | .003 | .32 | .11 | .003 |
| Valence x Arousal |  |  |  | -.08 | .21 | .708 | -.08 | .21 | .711 | -.08 | .21 | .695 |
| Child age |  |  |  |  |  |  | .29 | .05 | <.001 | .29 | .05 | <.001 |
| Child sex |  |  |  |  |  |  | -.11 | .11 | .33 | -.10 | .11 | .333 |
| Valence x age |  |  |  |  |  |  |  |  |  | -.06 | .07 | .373 |
| Arousal x age |  |  |  |  |  |  |  |  |  | .28 | .07 | <.001 |
| Valence x Arousal x age |  |  |  |  |  |  |  |  |  | .17 | .14 | .230 |
| Ordinal dependent variable: 0=incorrect, 1=valence recognition only, 2=exact recognition. Emotional valence: negative=-.5 and positive=.5. Arousal level: low=-.5 and high=.5. Child sex: female=-.5 and male=.5. Age was standardized. Results remain the same as Table 1 after controlling for random intercept by musical clip. | | | | | | | | | | | | |

| **Table S14.** Associations between CU traits, conduct problems, and music emotion recognition accuracy controlling for by-clip random effect | | | | | | |
| --- | --- | --- | --- | --- | --- | --- |
| *Predictor* | **Model 1** | | | **Model 2** | | |
|  | *B* | *SE* | *p* | *B* | *SE* | *p* |
| Emotional Valence | .05 | .10 | .640 | .04 | .10 | .701 |
| Arousal level | .32 | .11 | .003 | .33 | .10 | <002 |
| Valence x Arousal | -.08 | .21 | .719 | -.08 | .14 | .705 |
| Child age | .29 | .05 | <.001 | .29 | .05 | <.001 |
| Child sex | -.07 | .10 | .474 | -.08 | .10 | .469 |
| Child CU traits | -.18 | .06 | .004 | -.18 | .06 | .004 |
| Child CP | .03 | .06 | .617 | .03 | .06 | .608 |
| CU traits x Valence |  |  |  | -.14 | .07 | .044 |
| CU traits x Arousal |  |  |  | .15 | .07 | .041 |
| CU traits x Valence x Arousal |  |  |  | -.14 | .14 | .345 |
| CU=Callous-unemotional, CP=conduct problems; Ordinal outcome: 0=incorrect, 1=valence recognition only, 2=exact recognition. Emotional valence: negative=-.5 and positive=.5. Arousal level: low=-.5 and high=.5. Child sex: female=-.5 and male=.5. Age, CU traits and CP were standardized. Results remain the same as Table 1 after controlling for random intercept by musical clip. | | | | | | |

| **Table S15.** Bifactor model of the Inventory of Callous-Unemotional Traits (ICU) items to estimate a general CU traits factor score | | | | | | | |
| --- | --- | --- | --- | --- | --- | --- | --- |
|  | **General factor** | | | **Specific factors** | | | |
|  | *B* | *SE* | *p* |  | *B* | *SE* | *p* |
| 1. Expresses his/her feelings openly. | .47 | .08 | <.001 | **Callousness** |  |  |  |
| 2. Does not seem to know “right” from “wrong”. | .38 | .10 | <.001 | 2. Does not seem to know “right” from “wrong”. | .48 | .08 | <.001 |
| 4. Does not care who he/she hurts to get what he/she wants. | .67 | .07 | <.001 | 4. Does not care who he/she hurts to get what he/she wants. | .29 | .10 | .004 |
| 5. Feels bad or guilty when he/she has done something wrong. | .55 | .07 | <.001 | 7. Does not care about being on time. | .49 | .09 | <.001 |
| 7. Does not care about being on time. | .26 | .09 | .003 | 8. Is concerned about the feelings of others. | -.03 | .08 | .707 |
| 8. Is concerned about the feelings of others. | .82 | .04 | <.001 | 9. Does not care if he/she is in trouble. | .76 | .08 | <.001 |
| 9. Does not care if he/she is in trouble. | .25 | .10 | .011 | 11. Does not care about doing things well. | .75 | .08 | <.001 |
| 11. Does not care about doing things well. | .33 | .09 | .001 | 12. Seems very cold and uncaring. | .47 | .10 | .<.001 |
| 12. Seems very cold and uncaring. | .80 | .11 | <.001 | 18. Shows no remorse | .46 | .10 | <.001 |
| 13. Easily admits to being wrong. | .55 | .10 | <.001 | 20. Does not put the time into doing things well. | .51 | .09 | <.001 |
| 14. It is easy to tell how he/she is feeling. | .54 | .08 | <.001 | 21. The feelings of others are unimportant to him/her. | .37 | .09 | <.001 |
| 15. Always tries his/her best. | .65 | .08 | <.001 | **Uncaring** |  |  |  |
| 16. Apologizes to persons he/she has hurt. | .67 | .06 | <.001 | 5. Feels bad or guilty when he/she has done something wrong. | .07 | .16 | .671 |
| 17. Tries not to hurt others’ feelings. | .79 | .04 | <.001 | 13. Easily admits to being wrong. | .39 | .29 | .183 |
| 18. Shows no remorse | .50 | .08 | <.001 | 16. Apologizes to persons he/she has hurt. | .29 | .25 | .247 |
| 19. Is very expressive and emotional. | .20 | .09 | .028 | 23. Works hard on everything. | -.04 | .15 | .778 |
| 20. Does put the time into doing things well. | .25 | .10 | .009 | 24. Does things to make others feel good. | -.21 | .24 | .371 |
| 21. Feelings of others are unimportant | .55 | .07 | <.001 | **Unemotional** |  |  |  |
| 22. Hides his/her feelings from others. | .29 | .12 | .019 | 1. Expresses his/her feelings openly. | .58 | .08 | <.001 |
| 23. Works hard on everything. | .38 | .09 | <.001 | 14. It is easy to tell how he/she is feeling. | .62 | .08 | <.001 |
| 24. Does things to make others feel good. | .81 | .05 | <.001 | 19. Is very expressive and emotional. | .68 | .09 | <.001 |
|  |  |  |  | 22. Hides his/her feelings from others. | .46 | .12 | <.001 |
| CU=Callous Unemotional. ICU=Inventory of Callous-Unemotional Traits. Χ^2^(166)=245.16, p<.001, CFI=.94, TLI=.92, RMSEA=.054, SRMR=.093. Using confirmatory factor analysis, we tested a bifactor model whereby all items were specified to load onto a general CU traits factor, while simultaneously loading onto their requisite specific factor (i.e., callousness, uncaring, unemotional). General factor scores were extracted for each participant for use in subsequent analyses. Note that items 15 (“Always tries his/her best.”) and 17 (“Tries not to hurt others’ feelings”) were only specified to have general factor variance. | | | | | | | |

| **Table S16.** Latent factor model to extract general CP score from parent report of the SDQ | | | |
| --- | --- | --- | --- |
| Item | **General factor** | | |
|  | *B* | *SE* | *P* |
| 5. Often loses temper | .48 | .11 | <.001 |
| 7. Generally well behaved, usually does what adults request (reverse coded) | .78 | .11 | <.001 |
| 12. Often fights with other children or bullies them | .81 | .14 | <.001 |
| 18. Often lies or cheats | .85 | .12 | <.001 |
| 22. Steals from home, school or elsewhere | .10 | .16 | .528 |
| CP=Conduct Problems, SDQ=Strengths and Difficulties Questionnaire Χ^2(^5)=3.34, p=.647, CFI= 1.00, TLI=1.00, RMSEA=.000, SRMR=.064. Using confirmatory factor analysis, all items were specified to load onto a single CP factor. Factor scores were extracted for each participant for use in subsequent analyses. | | | |

| **Table 17.** Associations between the sum scores of CU traits, conduct problems, and music emotion recognition accuracy | | | | | | |
| --- | --- | --- | --- | --- | --- | --- |
| *Predictor* | **Model 1** | | | **Model 2** | | |
|  | *B* | *SE* | *p* | *B* | *SE* | *p* |
| Emotional Valence | .05 | .07 | .497 | .05 | .07 | .506 |
| Arousal level | .31 | .07 | <.001 | .31 | .07 | <.001 |
| Valence x Arousal | -.08 | .14 | .598 | -.07 | .04 | .605 |
| Child age | .29 | .05 | <.001 | .29 | .05 | <.001 |
| Child sex | -.09 | .11 | .415 | -.09 | .11 | .410 |
| Child CU traits | -.13 | .07 | .057 | -.13 | .07 | .058 |
| Child CP | .06 | .07 | .367 | .06 | .07 | .368 |
| CU traits x Valence |  |  |  | -.13 | .07 | .079 |
| CU traits x Arousal |  |  |  | .09 | .07 | .204 |
| CU traits x Valence x Arousal |  |  |  | -.15 | .15 | .307 |
| CU=Callous-unemotional, CP=conduct problems; Ordinal outcome: 0=incorrect, 1=valence matching, 2=exact recognition. Emotional valence: negative=-.5 and positive=.5. Arousal level: low=-.5 and high=.5. Child sex: female=-.5 and male=.5. Age. CU traits and CP were standardized. | | | | | | |

| **Table S18.** Mixed-effect logistic regression analyses examining whether the emotional valence and arousal level of sound predict the accuracy of emotion recognition from music. | | | | | | | | | | | | |
| --- | --- | --- | --- | --- | --- | --- | --- | --- | --- | --- | --- | --- |
| *Predictor* | **Model 1** | | | **Model 2** | | | **Model 3** | | | **Model 4** | | |
|  | *B* | *SE* | *p* | *B* | *SE* | *p* | *B* | *SE* | *p* | *B* | *SE* | *p* |
| Emotional Valence | .06 | .08 | .483 | .06 | .08 | .468 | .06 | .08 | .473 | .06 | .08 | .417 |
| Arousal level | .38 | .08 | <.001 | .38 | .08 | <.001 | .38 | .08 | <.001 | .36 | .08 | <.001 |
| Valence x Arousal |  |  |  | -.09 | .16 | .597 | -.09 | .16 | .599 | -.10 | .16 | .549 |
| Child age |  |  |  |  |  |  | .33 | .06 | <.001 | .33 | .06 | <.001 |
| Child sex |  |  |  |  |  |  | -.09 | .12 | .441 | -.09 | .12 | .441 |
| Valence x age |  |  |  |  |  |  |  |  |  | -.13 | .08 | .123 |
| Arousal x age |  |  |  |  |  |  |  |  |  | .29 | .08 | <.001 |
| Valence x Arousal x age |  |  |  |  |  |  |  |  |  | .22 | .16 | .190 |
| Dichotomous dependent variable: 0=incorrect, 1=exact recognition (partial recognition of correct valence matching was coded as 0). Emotional valence: negative=-0.5 and positive=0.5. Arousal level: low=-0.5 and high=0.5. Child sex=-0.5 and male=0.5. Age was standardized. | | | | | | | | | | | | |

| **Table S19.** Mixed-effect logistic regression analyses examining whether CU traits and conduct problems predict the accuracy of emotion recognition from music. | | | | | | |
| --- | --- | --- | --- | --- | --- | --- |
| *Predictor* | **Model 1** | | | **Model 2** | | |
|  | *B* | *SE* | *p* | *B* | *SE* | *p* |
| Emotional Valence | .06 | .08 | .473 | .05 | .08 | .534 |
| Arousal level | .38 | .08 | <.001 | .39 | .08 | <.001 |
| Valence x Arousal | -.09 | .16 | .600 | -.10 | .16 | .559 |
| Child age | .33 | .06 | <.001 | .33 | .06 | <.001 |
| Child sex | -.06 | .12 | .612 | -.06 | .12 | .617 |
| Child CU traits | -.17 | .07 | .013 | -.17 | .07 | .011 |
| Child CP | .04 | .07 | .590 | .04 | .07 | .589 |
| CU traits x Valence |  |  |  | -.12 | .08 | .149 |
| CU traits x Arousal |  |  |  | .13 | .08 | .106 |
| CU traits x Valence x Arousal |  |  |  | -.18 | .16 | .67 |
| CU=Callous-unemotional, CP=conduct problems. Dichotomous dependent variable: 0=incorrect, 1=exact recognition (partial recognition of correct valence matching was coded as 0). Emotional valence: negative=-0.5 and positive=0.5. Arousal level: low=-0.5 and high=0.5. Child sex=-0.5 and male=0.5. Age, CU traits, and CP were standardized. | | | | | | |

**Figure S1.** Response options for the Music Emotion Listening Task (MELT)

| **a.**  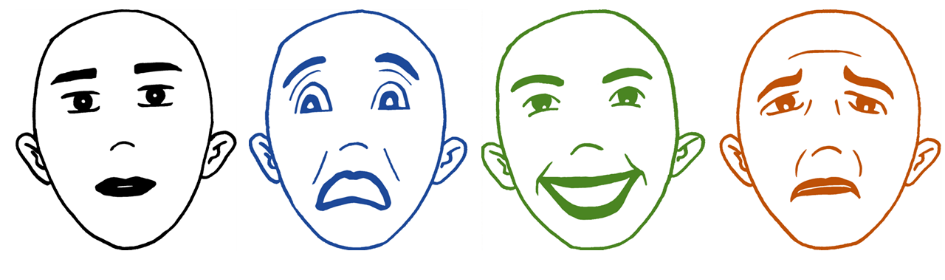 | | 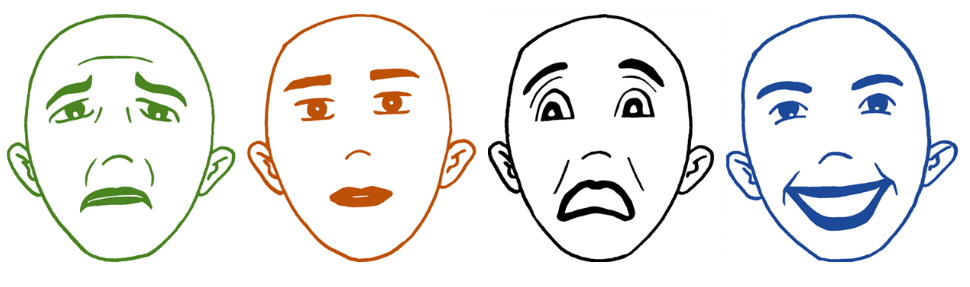**b.** |
| --- | --- | --- |
| **c.**  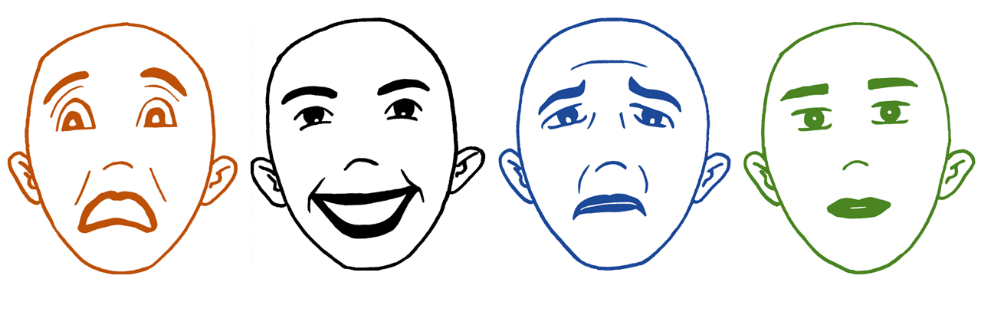 | 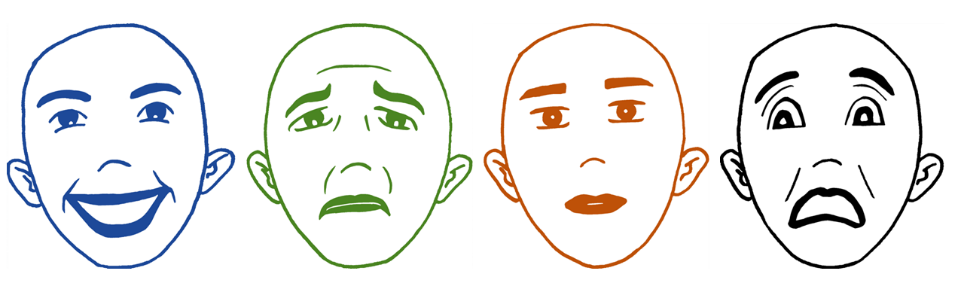**d.** | |
| *Note.* Children were presented with 20 music clips randomized across participants. Children had to identify which face felt a given emotion, answering by pointing to the face or naming the color of the face (black, red, green, and blue). Before the task, the research assistant reinforced the response options by naming the colors and emotions together (e.g., green is happy). Children were told that they would hear different music clips and would say which face matched the music by pointing to the face or naming its color. There was one practice trial. The colors and order of the face response options were fixed within participants (i.e., same order of faces depicted in the same colors each time) but colors/order was pseudorandomized between participants | | |

**Figure S2.** Children showed above chance accuracy in detecting the emotion conveyed by music

| 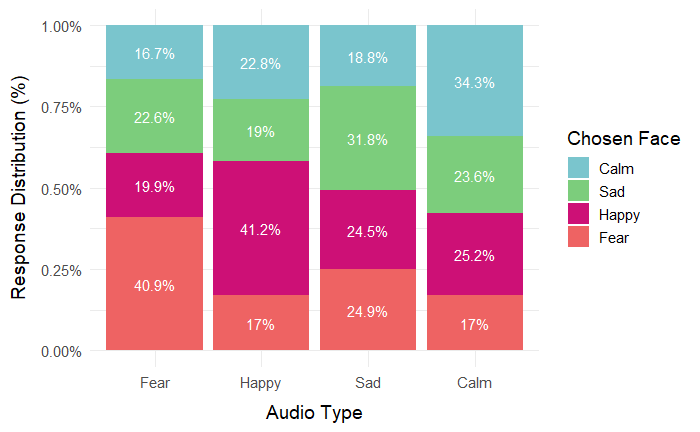 |
| --- |
| *Note.* For each emotion, children’s most frequent response was the correct emotion, the second most frequent response was the emotion that matched in valence to the correct response option. |

**Figure S3.** Children showed increasing accuracy in detecting the emotion conveyed by music with increasing age

| 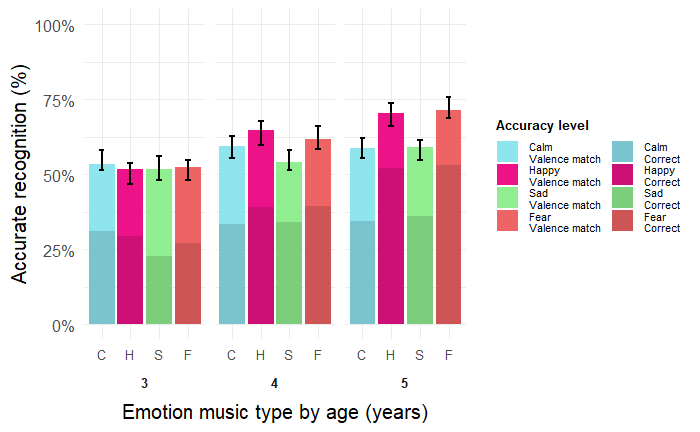 |
| --- |
| *Note.* Children show better emotion recognition in music with age. The increase for both fear and happy emotions recognition is greater than calm. While the increase in recognition of sadness compared to calm was not significant. |

**Figure S4.** Children are better at recognizing emotions at music in key major

| 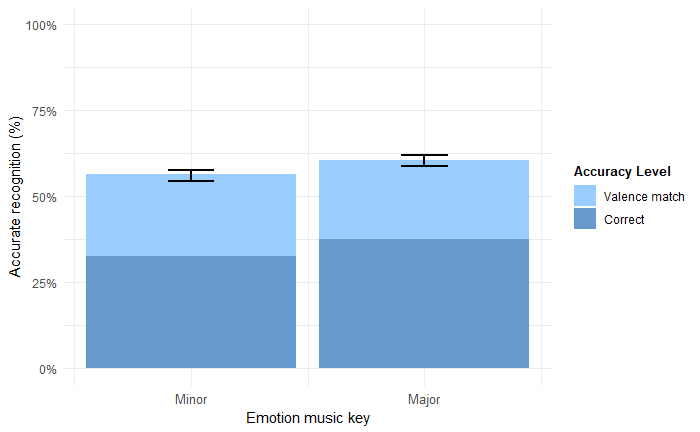 |
| --- |
| *Note.* Children show better emotion recognition in music for music clip in Major key. |

**Figure S5.** The association between CU traits and music emotion recognition accuracy varies by emotion type

| 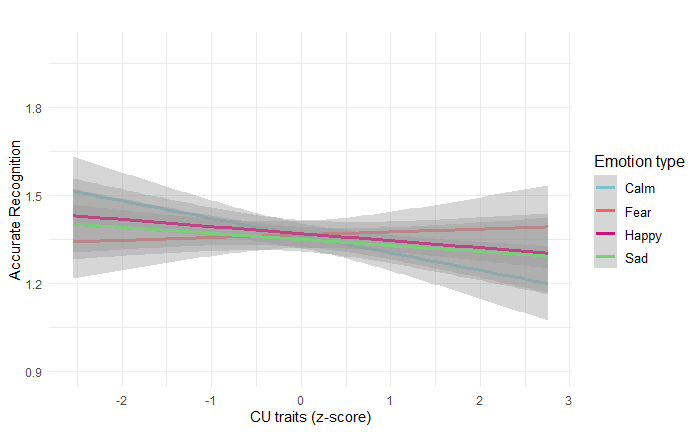 |
| --- |
| CU=Callous Unemotional. Higher CU traits correlated with fewer correct trials for calm (r=-.27, p<.01) and sad (r=-.17, p=.04), and unrelated to correct trials for happy (r=-.16, p=.06) or fearful music (r=.04, p=.63). |

**References**

Ray, J. V., & Frick, P. J. (2020). Assessing Callous-Unemotional Traits Using the Total Score from the Inventory of Callous-Unemotional Traits: A Meta-Analysis. *Journal of Clinical Child & Adolescent Psychology*, *49*(2), 190–199. https://doi.org/10.1080/15374416.2018.1504297

Rodriguez, A., Reise, S. P., & Haviland, M. G. (2016). Evaluating bifactor models: Calculating and interpreting statistical indices. *Psychological Methods*, *21*(2), 137–150. https://doi.org/10.1037/met0000045

Wagner, H. L. (1993). On measuring performance in category judgment studies of nonverbal behavior. *Journal of nonverbal behavior, 17*, 3-28.
